# *De novo* retrotransposon insertion into the *FGFR1* gene in a boy with congenital hypogonadotropic hypogonadism: a case report

**DOI:** 10.3389/fendo.2025.1565316

**Published:** 2025-06-18

**Authors:** Kentaro Sawano, Keisuke Nagasaki, Erina Suzuki, Yasuko Ogiwara, Ikuko Kageyama, Maki Fukami, Yoko Kuroki

**Affiliations:** ^1^ Division of Pediatrics, Department of Homeostatic Regulation and Development, Niigata University Graduate School of Medical and Dental Sciences, Niigata, Japan; ^2^ Department of Molecular Endocrinology, National Research Institute for Child Health and Development, Tokyo, Japan; ^3^ Division of Diversity Research, National Research Institute for Child Health and Development, Tokyo, Japan; ^4^ Department of Genome Medicine, National Research Institute for Child Health and Development, Tokyo, Japan; ^5^ Division of Collaborative Research, National Research Institute for Child Health and Development, Tokyo, Japan

**Keywords:** Alu element, FGFR1, hypogonadotropic hypogonadism, long-read NGS, retrotransposon

## Abstract

Congenital hypogonadotropic hypogonadism (CHH) is a rare endocrine disorder characterized by gonadal dysfunction attributed to impaired gonadotropin secretion. CHH is associated with approximately 60 genes including *FGFR1*. Nevertheless, the nucleotide variants of these genes are only related to less than half of the cases. Herein, we report a case of CHH caused by a novel mechanism. A 6-year-old boy presented with hypomasculinized genitalia, hyposmia, and syndactyly. Endocrine examinations showed impaired gonadotropin secretion. Short-read next-generation sequencing (NGS) identified the absence of mutations in the major causative genes for CHH. However, it detected an accumulation of discordant and split reads in a genomic region within the *FGFR1* gene. Array-based comparative genomic hybridization did not detect copy-number abnormalities. Targeted long-read NGS and Sanger sequencing identified a *de novo* 333-bp insertion in exon 9 of the *FGFR1* gene. A similarity search revealed that the insertion was an Alu element. This insertion caused a frameshift and resulted in premature termination (p. His409fsTer31). Further, it had several hallmarks of retrotransposition such as target site duplication, an endonuclease cleavage site-like motif, and a poly-A tail. The study results broadened the genetic basis of CHH that considered retrotransposon insertions. Importantly, this case emphasizes the need for additional genomic analyses in patients with CHH who had negative results on short-read NGS and array-based comparative genomic hybridization.

## 1 Introduction

Congenital hypogonadotropic hypogonadism (CHH) is a rare endocrine disorder characterized by gonadal dysfunction attributed to the impaired secretion of follicle-stimulating hormone (FSH) and luteinizing hormone (LH) from the pituitary gland (1). CHH typically causes hypomasculinized external genitalia in male neonates and delayed puberty in children of both sexes (1). Patients with CHH frequently exhibit additional clinical features such as anosmia/hyposmia and skeletal abnormalities (2–4).

CHH is a genetically heterogeneous condition. This disorder is associated with approximately 60 genes, including *FGFR1* (5–7). The most common pathogenic variants in these genes were single nucleotide substitutions and small indels. Meanwhile, the structural variants, such as deletions, insertions, and translocations, accounted for a small percentage of the cases (1). Based on a previous study, systematic mutation screening using short-read next-generation sequencing (NGS) and genome-wide copy-number analysis with array-based comparative genomic hybridization (CGH) identified pathogenic variants in less than half of patients with CHH (8). Therefore, unrecognized genetic abnormalities play a significant role in the development of CHH. In this context, *de novo* retrotransposon insertion into the genome is considered a rare cause of various congenital disorders (9). Nevertheless, it is not linked to CHH. Herein, we report the first case of CHH caused by retrotransposon insertion.

## 2 Case description

**Figure 1:**
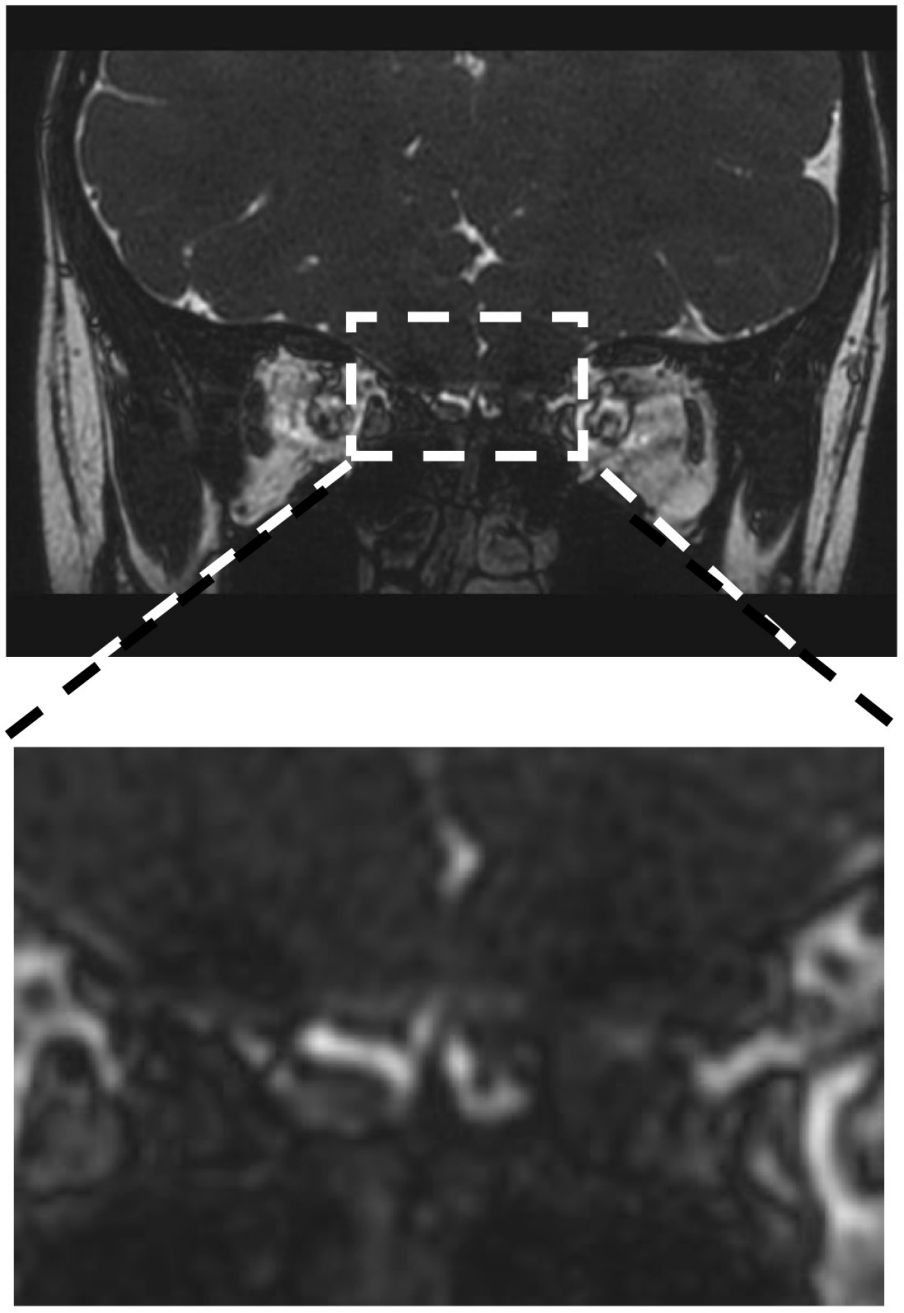
Clinical findings of the patient. Magnetic resonance imaging showed hypoplasia of the left olfactory groove and olfactory bulb.

The patient was a 6-year-old boy. **Table 1** shows the clinical characteristics of the patient. He was the first child of a nonconsanguineous Japanese couple. His parents had normal clinical characteristics. The patient was born at 38 weeks of gestation via cesarean section because his mother experienced hemolysis, elevated liver enzymes, and low platelets (HELLP) syndrome. His birth weight and height were 1,668 (−3.8 standard deviation [SD]) g and 43 (−2.4 SD) cm, respectively. At birth, he presented with micropenis (stretched penile length: 20 mm), bilateral cryptorchism, and 3/4 syndactyly of the right foot. Endocrine examinations at 3 months of age during minipuberty revealed low LH, FSH, and testosterone levels. The gonadotropin-releasing hormone stimulation test performed at 7 months of age revealed basal/peak LH and FSH levels at <0.2/2.3 and 1.1/24.8 IU/L, respectively. The peak LH and FSH levels were within the normal range, which indicated hypogonadotropic hypogonadism. Magnetic resonance imaging showed hypoplasia of the left olfactory groove and olfactory bulb (**Figure 1**). Thus, the patient was clinically diagnosed with CHH. He underwent orchidopexy at 2.1 years of age. Repeated endocrine examinations during childhood invariably showed low blood gonadotropin and testosterone levels. At 5.4 years of age, he had standard height and weight. His penile size was 35 (−1.8 SD) mm. The testes, with a volume of 1.0 mL, were palpable in the scrotum. Olfactory testing with a T&T olfactogram revealed mild olfactory loss. In particular, the right olfactory bulb had a normal function. However, olfactory perception was not detected on the left side. This finding was in accordance with the magnetic resonance imaging results.

**Table 1:** Serum hormone levels of the patient.

Age at examination	Luteinizing hormone level (IU/L)		Follicle-stimulating hormone level (IU/L)		Testosterone level (nmol/L)	
	Patient	Reference intervals	Patient	Reference intervals	Patient	Reference intervals
0 years, 3 months	<0.2	0.62–4.08	<1.0	0.41–3.02	0.13	0.69–7.60
0 years, 6 months	<0.2	0.10–8.06	1.0	0.19–3.63	<0.12	0.42–5.77
1 years, 7 months	<0.2	0.02–0.15	<1.0	0.38–1.11	<0.12	<0.35
2 years, 8 months	<0.2	0.02–0.15	<1.0	0.38–1.11	0.12	<0.35
3 years, 8 months	<0.2	0.02–0.15	<1.0	0.38–1.11	<0.12	<0.35
5 years, 4 months	<0.2	0.02–0.15	<1.0	0.38–1.11	0.22	<0.35

The reference intervals for each hormone during infancy are based on previously reported data (10, 11). The reference ranges for hormone levels after the age of 1 year are based on the reference levels listed in the Japanese Society of Endocrinology (12) and the textbook of endocrinology (13).

## 3 Molecular analyses

**Figure 2:**
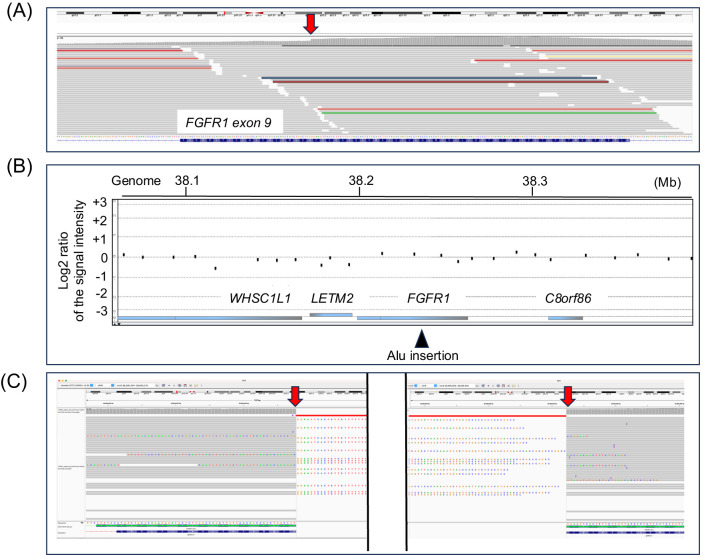
Representative results of the molecular analyses. **(A)** The results of short-read next-generation sequencing (NGS). Several discordant and split reads were identified in a genomic region within the FGFR1 gene. **(B)** The results of array-based comparative genomic hybridization. No copy-number variations were detected in the genomic region around the FGFR1 gene. **(C)** The results of the long-read NGS. The findings suggested a 333-bp insertion in exon 9. The arrowheads depict the insertion site.

Genomic DNA samples were obtained from the peripheral leukocytes of the patient and his parents. The DNA sample of the patient was subjected to mutation screening of the major causative genes for CHH (*ANOS1*, *CHD7*, *FGF8*, *FGFR1*, *GNRH1*, *GNRHR*, *IGSF1*, *KISS1*, *KISS1R*, *MKRN3, PROK2*, *PROKR2*, *SOX10*, *TAC3*, *TACR3*, and *WDR11*) (5–7). Short-read NGS was performed at the Kazusa DNA Research Institute (Chiba, Japan). The variants were analyzed, as described in a previous study (14). Results showed the absence of pathogenic nucleotide variants in the coding region of the 16 genes. However, the graphic visualization of the NGS data using the Integrative Genomics Viewer (https://igv.org) revealed an accumulation of discordant reads and split reads in a genomic region within the *FGFR1* gene (**Figure 2A**). These results indicated the presence of a structural variant in this region. Thus, CGH analysis was performed using a catalog array (Agilent Technologies, CA, the USA). However, copy-number variations were not identified in the genome of the patient (**Figure 2B**).

**Figure 3:**
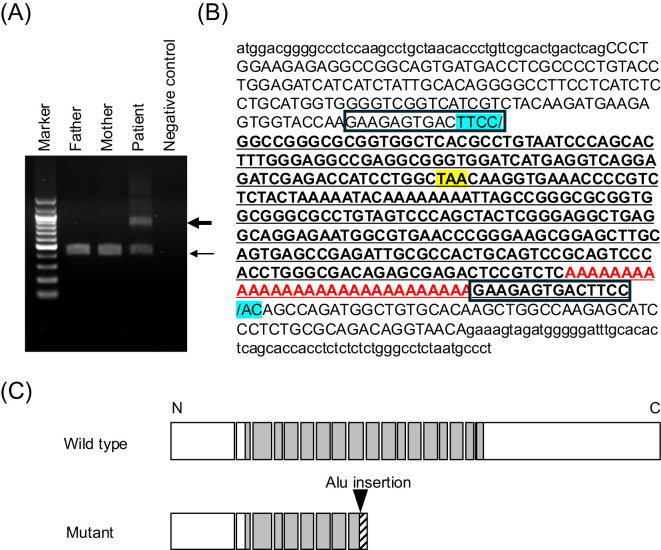
Characterization of the Alu insertion. **(A)** PCR analysis of exon 9 of the FGFR1 gene in the patient and his parents. The thin arrow indicates normal PCR products (422 bp). Meanwhile, the thick arrow denotes aberrant PCR products with the Alu insertion (755 bp). The aberrant PCR products were only obtained from the patient. **(B)** The sequences flanking the Alu insertion. Nucleotides in exon 9 are written in uppercase. The inserted sequence is underlined. The GAAGAGTGACTTCC sequences represent the target site duplication characteristic for retrotransposition. The inserted sequence containing the endonuclease cleavage site-like motif (TTCC/AC) is marked in light blue and the poly-A tail in red. **(C)** The predicted structure of the mutant protein. The Alu insertion was predicted to cause a frameshift and result in premature termination (p. His409fsTer31).

Then, targeted long-read sequencing was conducted using the patient’s DNA sample (Oxford Nanopore Technologies, Oxford, England). An approximately 100-kb genomic interval harboring *FGFR1* (T2T-CHM13/hs1, chr8:38,658,107–38,758,601) was subjected to adaptive sampling, as reported in a previous study (15, 16). Results showed a heterozygous insertion of an approximately 330-bp DNA fragment in exon 9 of the *FGFR1* gene (**Figure 2C**). Genomic region encompassing the insertion was PCR-amplified, and the PCR products were sequenced (**Figure 3A**). The insertion was found to be a 333-bp fragment (**Figure 3B**). A sequence similarity search using RepeatMasker (https://www.repeatmasker.org) revealed that the inserted sequence was an Alu element. The inserted sequence had known hallmarks of retrotransposition such as a target site duplication, an endonuclease cleavage site-like motif (TTCC/AC) at the breakpoints, and a poly-A tail (**Figure 3B**) (17, 18). This insertion has not been reported in patients with CHH and is absent from the gnomAD SV database (https://gnomad.broadinstitute.org/). This insertion was predicted to cause a frameshift from the 409th codon of the FGFR1 gene and result in premature termination at the 440th codon (p. His409fsTer31) (**Figure 3C**). PCR using the DNA samples of the patient’s parents yielded no products encompassing the insertion. Therefore, the insertion of the patient was a *de novo* variant (**Figure 3A**).

## 4 Discussion

In the current case, an unreported 333-bp insertion in the *FGFR1* gene was found in a boy with congenital hypogonadotropic hypogonadism. The patient exhibited CHH, hyposmia, and syndactyly, which are common features of *FGFR1* haploinsufficiency (19, 20). The patient presented with severe intrauterine growth failure, which is rare in patients with FGFR1 abnormalities. However, this phenotype can be attributed to HELLP syndrome (21, 22). Based on these results, the insertion of the patient destroyed the function of the *FGFR1* gene on one allele. Indeed, the mutated mRNA satisfied the condition of nonsense-mediated mRNA decay (23). This case emphasizes the importance of submicroscopic structural variants in the etiology of CHH.

In this case, the insertion was an Alu element. This insertion was a *de novo* variant, and it exhibited several hallmarks of retrotransposition such as target site duplication of 8–18 bp, the endonuclease cleavage site-like motif, and a poly-A tail. Alu is a primate-specific retrotransposon that accounts for approximately 10% of the human genome (24, 25). Retrotransposition of Alu and other repetitive elements is a common mechanism during gametogenesis or early embryogenesis. The rate of germline Alu retrotransposition is as high as 1 in 40 births (26). Retrotransposon insertions in the germline predominantly occur in intergenic or heterochromatin regions and usually permit a normal phenotype (26). However, these insertions can disrupt the coding regions or cis-regulatory elements, thereby causing genetic disorders (27). To date, patients with various monogenic disorders have presented with retrotransposon insertions into coding regions or regulatory elements (9, 28). To the best of our knowledge, this is the first case of CHH caused by retrotransposon insertion. Importantly, the insertion was not identified by short-read NGS. Nevertheless, the accumulation of split and discordant reads indicated the presence of a structural variant in the *FGFR1 gene*. Further, the insertion was overlooked by the array-based CGH. This can be explained by the fact that the probes of the custom CGH arrays are designed primarily in single-copy regions. Hence, retrotransposons may be hidden in other patients with CHH of unknown etiology.

In conclusion, the study results broadened the genetic basis of CHH that includes retrotransposition insertion. Based on this case, patients with CHH who have negative results for short-read NGS and array-based CGH require additional genomic analyses.

## Data Availability

The raw data supporting the conclusions of this article will be made available by the authors, without undue reservation.
